# Changes in echocardiographic parameters and strain and outcomes following mitral valve repair: insight from new mitral regurgitation classifications

**DOI:** 10.3389/fcvm.2025.1666071

**Published:** 2026-01-12

**Authors:** Roslan Aslannif, Si Ling Soh, Yee Sin Tey, Am Haris Norhaliza, Teo Jassie, Lynne Murray Rowina, Kee Soon Chong, Kian Boon Wong, Dillon Jeswant, Mohamed Yunus Alwi, Mohd Ghazi Azmee

**Affiliations:** 1 Cardiology Department, Institut Jantung Negara, Kuala Lumpur, Malaysia; 2 Cardiothoracic Department, Institut Jantung Negara, Kuala Lumpur, Malaysia

**Keywords:** cardiac function, echocardiographic parameters, mitral regurgitation, mitral valve repair, outcomes

## Abstract

**Introduction:**

Mitral valve repair is a cornerstone treatment for mitral regurgitation (MR), but outcomes across various etiologies remain incompletely understood. Therefore, this study aimed to comprehensively assess the outcomes of mitral valve repair across different MR etiologies, including primary MR, left ventricle functional MR (LVFMR), ischemic MR, left atrial functional MR (LAFMR), and rheumatic MR, by examining in-hospital, short-term, and medium-term outcomes, as well as the change in cardiac function through echocardiographic parameters and strain analysis.

**Methods:**

This retrospective study included 911 patients who underwent mitral valve repair at a single center from 2015 to 2021. Echocardiographic and strain analyses were performed preoperatively and at 6 months post-surgery. Mortality outcomes were assessed at in-hospital and at 6, 12, and 51 months postoperatively.

**Results:**

There were significant differences in outcomes across MR etiologies. The primary and rheumatic groups demonstrated the lowest mortality rates, whereas the LVFMR, LAFMR, and ischemic groups exhibited markedly higher mortality, both in the early postoperative period and during long-term follow-up. Echocardiographic analysis showed improvements in cardiac dimensions and volumes, but concurrent declines in myocardial function, particularly in ejection fraction, global longitudinal strain, and left atrial reservoir strain.

**Discussion:**

This study highlights the importance of etiology-specific risk stratification. While mitral valve repair effectively reduces MR, myocardial recovery may be delayed or insufficient in certain subgroups, especially those with pre-existing myocardial dysfunction. Tailored therapeutic strategies that address the multifactorial nature of functional and ischemic MR are warranted to improve long-term outcomes.

## 1 Introduction

Mitral valve (MV) repair has emerged as a cornerstone treatment for mitral regurgitation (MR) since Bailey et al. introduced a pioneering approach in 1951 using left thoracotomy and external compression of the mitral annulus (1). This foundational method was further refined in 1957 by Lillehei et al., who performed the first direct suture annuloplasty using a cardiopulmonary bypass (2). Although MV repair has been proven to be effective in reducing or eliminating MR, its outcomes across various etiologies remain unclear. This gap is particularly substantial, given the diverse pathophysiological mechanisms of MR.

Contemporary classification systems for MR etiology have improved our ability to systematically approach these conditions and have created an opportunity to evaluate outcome patterns specific to each etiologic subgroup. These systems categorize MR into conceptually straightforward groups based on underlying anatomic and imaging features, including leaflet abnormalities such as prolapse and/or flail and rheumatic changes, as well as left ventricular and left atrial dilatation (3–5). However, although hemodynamic changes can vary substantially among these different etiologies, comprehensive studies describing postsurgical echocardiographic changes in cardiac function and chamber dimensions remain limited. This gap is particularly evident for studies incorporating speckle-tracking strain analysis across multiple MR etiologies, with the last major echocardiographic study in this area published in 1994 (6).

Therefore, this study aimed to comprehensively assess the outcomes of MV repair across different MR etiologies by examining the in-hospital, short-term, and medium-term outcomes. Additionally, we investigated the evolution of cardiac function through detailed echocardiographic parameters and myocardial strain analysis at 6 months postoperatively, providing crucial insights into the differential patterns of cardiac remodeling specific to each MR etiology.

## 2 Materials and methods

### 2.1 Study design and population

This retrospective study included all adults (aged >18 years) who underwent MV repair at the Institut Jantung Negara in Kuala Lumpur, Malaysia, between January 1, 2015, and December 31, 2021. Patients younger than 18 years of age, those with congenital heart disease or infective endocarditis, and cases where post-repair echocardiograms were either unavailable or of insufficient image quality for analysis were excluded. All patients underwent baseline preoperative transthoracic echocardiography (Pre-TTE), intraoperative transesophageal echocardiography (Intra-TEE), and postoperative transthoracic echocardiography (Post-TTE) performed within 6 months of surgery. Demographic data, complications, and in-hospital mortality were obtained from medical records, whereas additional mortality data were collected from medical records and the Malaysia National Registry Census until January 31, 2025. Mortality outcomes were assessed at four time points: in-hospital and at 6, 12, and at median of 51 months (4.3 years, IQR 17.3,79.9)) post-intervention.

The ethics committee of the Institut Jantung Negara approved this study (approval number: IJNREC/665/2024).

### 2.2 MR etiologies

Pre-TTE, Intra-TEE, Post-TTE, and surgical reports were comprehensively analyzed to determine the underlying etiologies. The echocardiographic images are essential for complementing surgical reports, as they allow direct visualization of leaflet motion. They can demonstrate excessive motion (prolapse and/or flail), restricted opening and closure in rheumatic mitral regurgitation, tethering and tenting in ischemic and left ventricle functional mitral regurgitation, and a dilated left atrial annulus in left atrial functional mitral regurgitation. Primary MR is defined as MR resulting from excessive leaflet motion presenting as prolapse and/or flail. Left ventricular functional mitral regurgitation (LVFMR) is defined as MR that results from left ventricular dilatation rather than primary leaflet pathology. The ventricular remodeling produces a combination of leaflet tethering and tenting, with or without annular dilatation. LVFMR is observed either in patients without coronary artery disease (CAD) or in those with CAD when the ejection fraction (EF) is below 35%. In contrast, ischemic mitral regurgitation (ischemic MR) is defined as MR secondary to CAD and is typified by regional wall motion abnormalities and/or left ventricular dilation leading to tethering and tenting of the mitral valves, with an EF equal to or greater than 35%. All patients underwent coronary evaluations before surgery either with invasive coronary angiogram or Multislice Computed Tomography (MSCT) coronary assessments. Left atrial functional mitral regurgitation (LAFMR) is defined as MR resulting from left atrial dilation, which in turn causes annular dilatation, while the left ventricular size and EF remain normal. Most common cause of LAFMR is atrial fibrillation (AF) followed by heart failure with preserved ejection fraction (HFpEF). Rheumatic MR is attributed to rheumatic disease and is evident from the thickening and calcification of the mitral valves, resulting in restricted closure and/or pseudo-prolapse of the anterior MV leaflet. Pseudo-prolapse occurs in rheumatic mitral valves when the posterior leaflet is very short or even absent. This produces the appearance of anterior mitral leaflet prolapse during systole. Lastly, cases were classified as “others” when a confident etiological determination could not be made on the basis of the available clinical and imaging data.

### 2.3 Echocardiographic parameters

Pre-TTE and Post-TTE images were analyzed for comprehensive cardiac parameters. Left ventricular measurements included left ventricular EF, indexed end-diastolic and end-systolic volumes [left ventricular end-diastolic volume index (LVEDVi) and left ventricular end-systolic volume index (LVESVi), respectively], end-diastolic and end-systolic dimensions [left ventricular internal diameter in diastole (LVIDd) and left ventricular internal dimension, respectively], interventricular septal diameter, and posterior wall thickness diameter. Additional parameters assessed included the left atrial volume index (LAVI), measured using two-dimensional TTE in the apical 2-chamber and 4-chamber views with the biplane Simpson method, relative wall thickness, and tricuspid regurgitation maximum velocity. Myocardial deformation analysis was performed retrospectively using Philips Ultrasound Workspace software to evaluate multiple strain parameters, with a minimum frame rate of 60 Hz required. These parameters included global longitudinal strain (GLS), which required high-quality apical 4-chamber, 3-chamber, and 2-chamber views; left atrial reservoir strain (LArS), which measures left atrial expansion during left ventricular systole when it receives blood from the pulmonary veins; left atrial conduit strain (LAcS), which reflects left atrial function during early left ventricular diastole when blood flows passively from the left atrium to the left ventricle; and left atrial booster strain (LAbS), which assesses atrial contraction at the end of ventricular diastole. All left atrial strain measurements were obtained from the apical 4-chamber view, with zooming on the left atrium to avoid foreshortening. Finally, right ventricular free wall strain (RVFWS) was measured from an RV-focused apical view, excluding the interventricular septum to minimize left ventricular contribution.

### 2.4 Mitral regurgitation severity assessments

MR severity was assessed using preoperative transthoracic echocardiography. Visual assessment included evaluation of the width and neck of the regurgitant jet, its spatial extent, and its duration (holosystolic vs. early or late systolic). Semiquantitative parameters included pulmonary venous Doppler pattern, early diastolic mitral inflow velocity (E wave), and left ventricular and left atrial dimensions. In the majority of patients, MR severity was further quantified using the proximal isovelocity surface area (PISA) method, with an effective regurgitant orifice area (EROA) > 0.4 cm^2^ and regurgitant volume > 60 mL defining severe MR. All visual, semiquantitative, and quantitative parameters were integrated to derive a final grading of mild, moderate, or severe MR.

Two experienced echocardiographers independently graded MR severity; in cases of discrepancy, a third echocardiographer reviewed the study, and the final severity grade was determined by consensus. All echocardiographers were certified in adult echocardiography and routinely interpret echocardiograms as part of their daily clinical practice.

### 2.5 Statistical analysis

Demographic and baseline characteristics are summarized as mean with standard deviation or median with interquartile range for continuous variables and as frequency with percentage for categorical variables. Group comparisons were conducted using appropriate statistical tests, including the paired t-test or Wilcoxon ranked test (where applicable) for continuous variables and the chi-square or Fisher exact test for categorical variables (where applicable). Kaplan–Meier curves were used to estimate and visually represent the probability of survival from all-cause mortality following the surgical admission discharge. Censoring was appropriately applied in cases where patients were lost to follow-up or where mortality had not yet occurred by the end of the study. The log-rank test was used to compare survival curves between the subgroups. Statistical analyses were performed using SPSS Statistics (version 29 (IBM Corp., Armonk, NY, USA), with statistical significance set at *p* < 0.05.

## 3 Results

**Figure 1:**
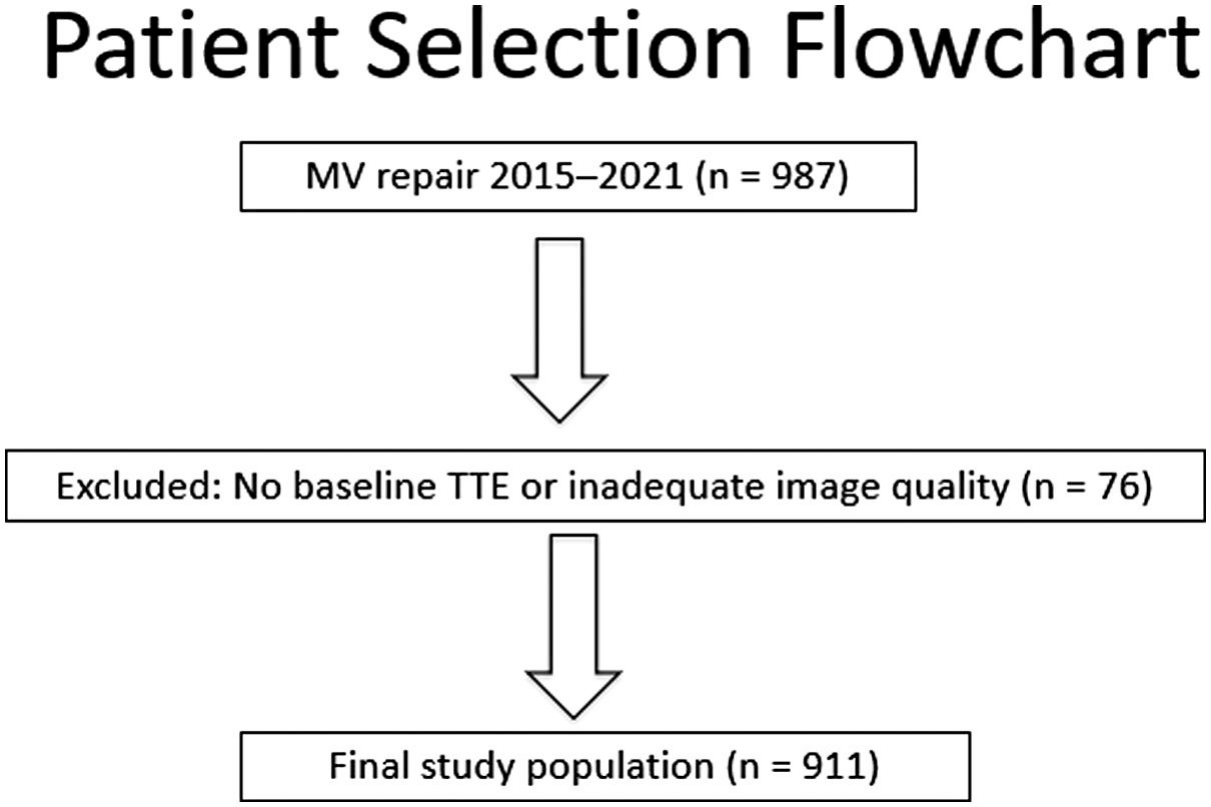
Patient selection flowchart. In total there are 987 patients that underwent MV repair from 2015 to 2021. 76 patients were excluded due to no baseline TTE or inadequate image quality. Final number included in the study is 911 patients. MV, mitral valves.

**Figure 2:**
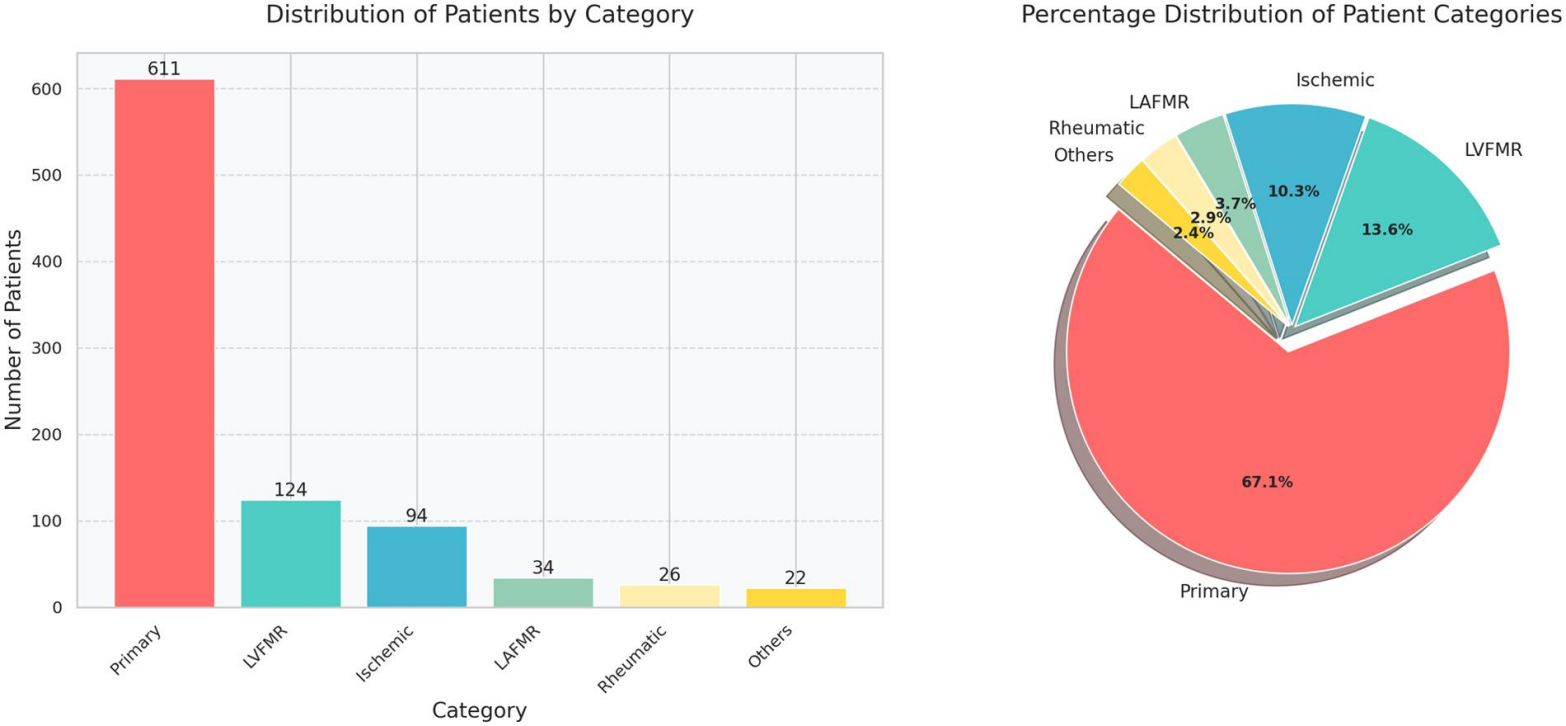
Distribution of patients across the subgroups. The highest number of patients are in the primary MR group followed by the LVFMR, ischemic, LAFMR, rheumatic, and others groups. LVFMR, left ventricle functional mitral regurgitation; LAFMR, left atrial functional mitral regurgitation; MR, mitral regurgitation.

A total of 987 patients had MV repair from 2015 to 2021.Out of these 911 patients were included in this study with 76 patients excluded due to no baseline TTE and inadequate quality TTE images to determine correct etiologies (Figure 1). The distribution of patients across the subgroups is shown in Figure 2. Patients' baseline characteristics are presented in Table 1.

**Table 1:** Baseline characteristics of patients according to different mechanisms of mitral regurgitation.

Variable		Total *N* = 991	Mechanism of MR					
			Primary (*N* = 611)	LVFMR (*N* = 124)	Ischemic (*N* = 94)	LAFMR (*N* = 34)	Rheumatic (*N* = 26)	Others (*N* = 22)
Age, y	Median (Q1, Q3)	56.4 (44.6, 63.6)	54.5 (43.4, 62.2)	59.6 (51.9, 65.0)	61.2 (56.1, 66.3)	64.6 (54.4, 69.0)	29.6 (21.7, 38.0)	47.2 (29.9, 59.5)
Preop BSA, m^2^	Median (Q1, Q3)	1.8 (1.7, 1.9)	1.8 (1.7, 1.9)	1.8 (1.7, 1.9)	1.8 (1.7, 1.9)	1.7 (1.7, 1.8)	1.7 (1.6, 1.8)	1.8 (1.7, 1.8)
Sex, *n* (%)	Female	292 (32.1)	191 (31.3)	31 (25.0)	23 (24.5)	15 (44.1)	22 (84.6)	10 (45.5)
	Male	619 (67.9)	420 (68.7)	93 (75.0)	71 (75.5)	19 (55.9)	4 (15.4)	12 (54.5)
Race, *n* (%)	Malay	442 (48.5)	293 (48.0)	64 (51.6)	47 (50.0)	11 (32.4)	16 (61.5)	11 (50.0)
	Chinese	302 (33.2)	212 (34.7)	37 (29.8)	26 (27.7)	19 (55.9)	3 (11.5)	5 (22.7)
	Indian	82 (9.0)	53 (8.7)	10 (8.1)	15 (16.0)	0	2 (7.7)	2 (9.1)
	Other Malaysians	32 (3.5)	20 (3.3)	6 (4.8)	1 (1.1)	2 (5.9)	1 (3.8)	2 (9.1)
	Foreigner	53 (5.8)	33 (5.4)	7 (5.6)	5 (5.3)	2 (5.9)	4 (15.4)	2 (9.1)
MR severity, *n* (%)	Mild	64 (7.0)	22 (3.6)	25 (20.2)	7 (7.4)	5 (14.7)	1 (3.8)	4 (18.2)
	Moderate	315 (34.6)	144 (23.6)	74 (59.7)	69 (73.4)	14 (41.2)	7 (26.9)	7 (31.8)
	Severe	532 (58.4)	445 (72.8)	25 (20.2)	18 (19.1)	15 (44.1)	18 (69.2)	11 (50.0)
NYHA class, *n* (%)	I/II	802 (89.8)	560 (93.6)	92 (75.4)	79 (85.9)	32 (94.1)	20 (80.0)	19 (86.4)
	III/IV	91 (10.2)	38 (6.4)	30 (24.6)	13 (14.1)	2 (5.9)	5 (20.0)	3 (13.6)
Comorbidity, *n* (%)	Diabetes mellitus	171 (18.8)	64 (10.5)	46 (37.1)	48 (51.1)	7 (20.6)	1 (3.8)	5 (22.7)
	Hypertension	451 (49.5)	266 (43.5)	78 (62.9)	77 (81.9)	18 (52.9)	3 (11.5)	9 (40.9)
	Hypercholesterolemia	409 (44.9)	227 (37.2)	81 (65.3)	74 (78.7)	13 (38.2)	5 (19.2)	9 (40.9)
	Neurological diseases	29 (3.2)	19 (3.1)	2 (1.6)	4 (4.3)	2 (5.9)	0	2 (9.1)
	Pulmonary diseases	55 (6.0)	28 (4.6)	10 (8.1)	10 (10.6)	2 (5.9)	1 (3.8)	4 (18.2)
	Chronic kidney disease	101 (11.1)	42 (6.9)	27 (21.8)	22 (23.4)	4 (11.8)	1 (3.8)	5 (22.7)
	Previous cardiac surgery	19 (2.1)	12 (2.0)	2 (1.6)	0	2 (5.9)	1 (3.8)	2 (9.1)
Smoking history	Current/Former smoker	226 (24.8)	135 (22.1)	45 (36.3)	33 (35.1)	5 (14.7)	3 (11.5)	5 (22.7)
eGFR, mL/min/1.73 m^2^	Median (Q1, Q3)	68.9 (54.8, 85.6)	72.0 (60.3, 87.2)	58.5 (49.7, 78.6)	56.7 (36.4, 72.5)	60.9 (44.2, 81.4)	100.3 (78.8, 122.0)	64.1 (28.6, 90.4)
Renal function category	Stage 1	145 (20.0)	105 (22.3)	14 (12.6)	5 (6.2)	5 (19.2)	11 (61.1)	5 (26.3)
	Stage 2	343 (47.3)	252 (53.6)	37 (33.3)	34 (42.0)	9 (34.6)	6 (33.3)	5 (26.3)
	Stage 3	202 (27.9)	108 (23.0)	51 (45.9)	26 (32.1)	12 (46.2)	1 (5.6)	4 (21.1)

MR, mitral regurgitation; Q1, quartile 1; Q3, quartile 3; Preop, preoperative; BSA, body surface area; NYHA, New York Heart Association; eGFR, estimated glomerular filtration rate.

Overall, the EF decreased significantly from 58.0% to 50.0% (*p* < 0.001) at 6 months postoperatively, accompanied by reductions in the LVESVi from 32.07 to 25.94 mL/m^2^ (*p* < 0.001) and LVEDVi from 75.80 to 53.64 mL/m^2^ (*p* < 0.001). The LAVI showed a marked decrease from 58.97 to 35.35 mL/m^2^ (*p* < 0.001). Paradoxically, these favorable hemodynamic changes, reflecting reduced chamber dimensions and volumes, were accompanied by a deterioration in contractile function, as evidenced by decreased GLS from 18.80% to 14.00% (*p* < 0.001) and reduced LArS from 25.9% to 20.3% (*p* < 0.001). Subgroup analysis revealed differential responses among patient categories, with the primary MR group demonstrating the most pronounced changes including EF decline from 61.0% to 52.0% (*p* < 0.001), substantial volumetric improvements (LVEDVi: from 74.49 to 51.61 mL/m^2^, *p* < 0.001; LAVI: from 61.20 to 33.49 mL/m^2^; *p* < 0.001), and significant contractile function deterioration (GLS: from 20.50% to 15.20%; *p* < 0.001). However, the LVFMR group was the only subgroup that showed functional improvement, with the EF increasing from 30.0% to 33.0% (*p* = 0.004), GLS improving from 8.80% to 9.40% (*p* = 0.817), and LArS increasing from 12.30% to 15.20% (*p* = 0.087), despite experiencing moderate reductions in the LVEDVi and LAVI. The ischemic group, while maintaining a stable EF (from 40.0% to 38.5%, *p* = 0.067), exhibited a statistically significant deterioration in strain parameters (GLS: from 12.10% to 9.60%, *p* < 0.001; LArS: from 17.45% to 14.5%; *p* < 0.001) along with modest volumetric reductions.

**Figure 3:**
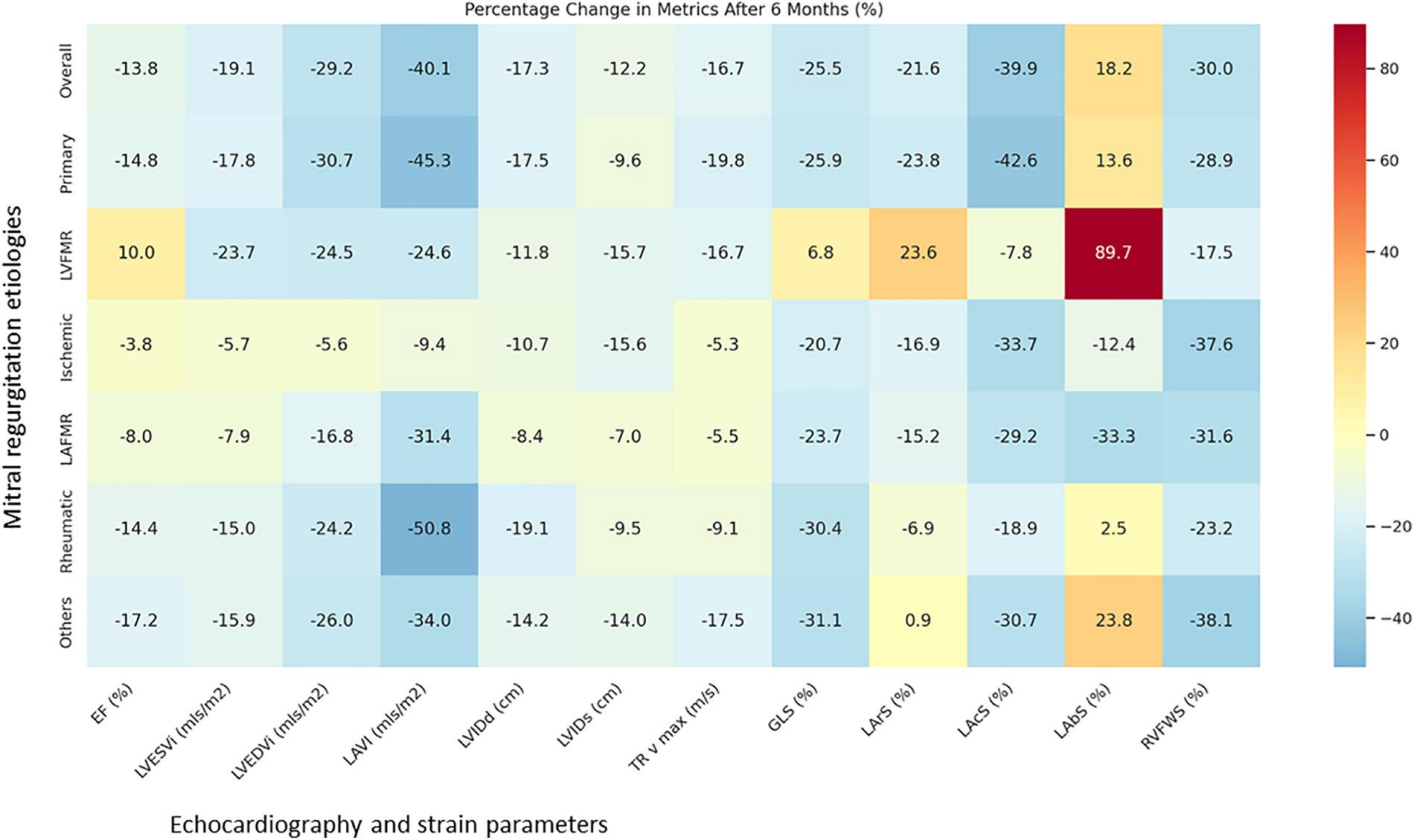
Percentage change in metrics after 6 months. The LV volume and dimensions are reduced in all groups. The EF, GLS and LArS also reduced except in LVFMR group. LV, left ventricular; EF, ejection fraction; GLS, global longitudinal strain; LArS, left atrial reservoir strain; LVESVi, left ventricular end-systolic volume index; LVEDVi, left ventricular end-diastolic volume index; LAVi, left atrial volume index; LVIDd, left ventricular internal dimension in diastole; LVIDs, left ventricular internal dimension in systole; Tr v max, tricuspid regurgitation maximum velocity; RVFWS, right ventricular free wall strain.

**Figure 4:**
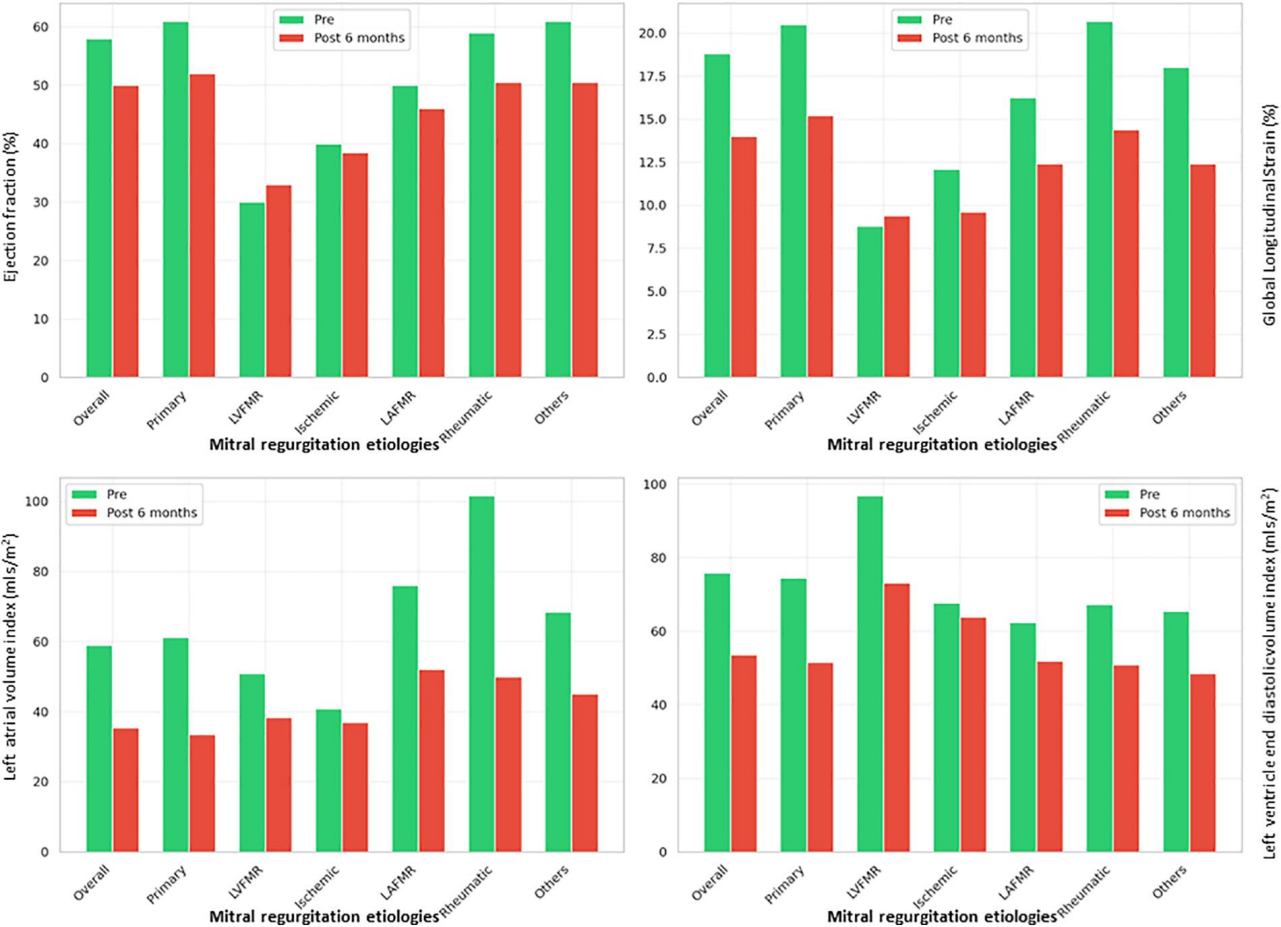
Changes in EF, GLS, LAVi, and LVEDVi in all groups. LAVi and LVEDVi are reduced in all groups, whereas only in LVFMR there are improvement in EF and GLS. EF, ejection fraction; GLS, global longitudinal strain; LAVi, left atrial volume index; LVEDVi, left ventricular end-diastolic volume index; LVFMR, left ventricle functional mitral regurgitation; Pre, preoperative; Post, postoperative.

The LAFMR subgroup showed modest EF decline with concurrent reductions in the LVEDVi, LAVI, GLS, and LArS. The Rheumatic MR group demonstrated marked structural remodeling similar to the primary MR group with highly significant reduction in EF, LV systolic and diastolic volumes, GLS and LArS. Similarly, the Others group exhibited comparable trends. In the overall cohort, additional classic echocardiographic parameters reinforced these findings, with the LVIDd and tricuspid regurgitation velocity maximum showing improvement, while LArS, LAcS, and RVFWS exhibited reductions. This pattern of improved hemodynamics coupled with reduced left ventricular, left atrial, and right ventricular contractile functions was consistent across all groups, with the notable exception of the LVFMR group, which demonstrated the smallest percentage change in RVFWS and showed improvements in the EF, GLS, and LArS, distinguishing it from all other subgroups (Table 2; Figures 3, 4).

**Table 2:** Echocardiography and strain analysis results before and after mitral valve repair according to different mechanisms of mitral regurgitation.

Variable			Total *N* = 991	Mechanisms of mitral regurgitation					
				Primary (*N* = 611)	LVFMR (*N* = 124)	Ischemic (*N* = 94)	LAFMR (*N* = 34)	Rheumatic (*N* = 26)	Others (*N* = 22)
LVEF, %	Pre	Median (Q1, Q3)	58.00 (46.00, 64.00)	61.00 (56.10, 65.00)	30.00 (26.00, 35.00)	40.00 (36.00, 44.93)	50.00 (44.00, 56.00)	59.00 (54.25, 63.30)	61.00 (57.05, 64.75)
	Post	Median (Q1, Q3)	50.00 (41.00, 56.00)	52.00 (46.90, 57.00)	33.00 (29.00, 40.00)	38.50 (34.50, 46.00)	46.00 (40.00, 52.00)	50.50 (43.00, 55.50)	50.50 (46.00, 58.75)
	*p*-value		<0.001*	<0.001*	0.004*	0.067	0.003*	0.001*	0.001*
LVESVi, mL/m^2^	Pre	Median (Q1, Q3)	32.07 (23.90, 45.05)	28.94 (22.50, 37.66)	65.00 (53.92, 80.00)	39.81 (29.95, 53.49)	32.00 (23.34, 40.98)	29.39 (18.08, 38.19)	26.70 (19.16, 37.80)
	Post	Median (Q1, Q3)	25.94 (19.65, 37.64)	23.79 (18.82, 31.58)	49.57 (33.56, 63.64)	37.54 (27.04, 45.50)	29.47 (19.26, 38.54)	24.99 (18.09, 32.10)	22.46 (15.52, 30.39)
	*p*-value		<0.001*	<0.001*	<0.001*	0.021*	0.491	0.304	0.227
LVEDVi, mL/m^2^	Pre	Median (Q1, Q3)	75.80 (59.45, 95.02)	74.49 (59.64, 92.07)	96.85 (77.81, 118.83)	67.67 (54.61, 86.62)	62.37 (51.67, 78.42)	67.24 (46.83, 83.46)	65.50 (43.40, 88.17)
	Post	Median (Q1, Q3)	53.64 (43.75, 68.16)	51.61 (42.60, 62.52)	73.17 (55.52, 93.23)	63.88 (47.98, 71.20)	51.92 (39.70, 69.43)	50.94 (39.13, 69.87)	48.47 (36.94, 57.37)
	*p*-value		<0.001*	<0.001*	<0.001*	0.001*	0.020*	0.003*	0.003*
LAVi, mL/m^2^	Pre	Median (Q1, Q3)	58.97 (43.28, 78.14)	61.20 (45.66, 80.88)	50.90 (39.98, 66.09)	40.80 (33.62, 54.82)	76.00 (55.86, 124.85)	101.64 (63.01, 131.35)	68.42 (38.81, 83.41)
	Post	Median (Q1, Q3)	35.35 (27.65, 46.25)	33.49 (26.56, 44.25)	38.84 (31.03, 46.22)	36.96 (30.19, 43.13)	52.16 (37.13, 86.35)	49.96 (36.94, 68.32)	45.14 (29.63, 63.26)
	*p*-value		<0.001*	<0.001*	<0.001*	0.004*	<0.001*	<0.001*	0.012*
LVIDd, cm	Pre	Median (Q1, Q3)	5.68 (5.15, 6.10)	5.60 (5.10, 6.06)	6.10 (5.70, 6.55)	5.60 (5.27, 6.07)	5.35 (4.86, 6.00)	5.54 (5.17, 5.95)	5.20 (4.53, 6.09)
	Post	Median (Q1, Q3)	4.70 (4.30, 5.26)	4.62 (4.24, 5.08)	5.38 (4.80, 5.99)	5.00 (4.50, 5.60)	4.90 (4.30, 5.43)	4.48 (3.88, 5.10)	4.46 (4.03, 4.75)
	*p*-value		<0.001*	<0.001*	<0.001*	<0.001*	<0.001*	<0.001*	0.004*
LVIDs, cm	Pre	Median (Q1, Q3)	3.76 (3.27, 4.41)	3.54 (3.11, 4.00)	5.11 (4.70, 5.60)	4.50 (3.90, 4.96)	3.87 (3.50, 4.44)	3.59 (3.30, 4.18)	3.51 (2.74, 4.03)
	Post	Median (Q1, Q3)	3.30 (2.95, 3.90)	3.20 (2.85, 3.60)	4.31 (3.73, 5.05)	3.80 (3.45, 4.63)	3.60 (3.10, 4.10)	3.25 (2.70, 3.85)	3.02 (2.57, 3.67)
	*p*-value		<0.001*	<0.001*	<0.001*	<0.001*	0.037*	0.009*	0.212
IVSd, cm	Pre	Median (Q1, Q3)	1.04 (0.90, 1.18)	1.06 (0.91, 1.19)	1.00 (0.90, 1.10)	1.04 (0.90, 1.18)	1.10 (0.94, 1.26)	0.91 (0.80, 1.00)	1.21 (0.86, 1.42)
	Post	Median (Q1, Q3)	1.00 (0.90, 1.19)	1.00 (0.90, 1.17)	1.00 (0.90, 1.20)	1.08 (0.90, 1.20)	1.10 (0.90, 1.20)	0.90 (0.80, 1.10)	1.13 (0.80, 1.30)
	*p*-value		0.002*	<0.001*	0.154	0.483	0.190	0.280	0.095
PWTd, cm	Pre	Median (Q1, Q3)	1.00 (0.87, 1.17)	1.02 (0.89, 1.18)	0.96 (0.80, 1.10)	0.99 (0.80, 1.12)	1.00 (0.85, 1.18)	0.96 (0.85, 1.13)	1.17 (1.01, 1.34)
	Post	Median (Q1, Q3)	1.00 (0.90, 1.17)	1.00 (0.90, 1.15)	1.08 (0.90, 1.20)	1.00 (0.90, 1.11)	1.00 (0.90, 1.20)	0.93 (0.80, 1.12)	1.10 (0.90, 1.20)
	*p*-value		0.393	0.536	<0.001*	0.245	0.473	0.808	0.027*
RWT	Pre	Median (Q1, Q3)	0.36 (0.30, 0.43)	0.37 (0.31, 0.44)	0.31 (0.26, 0.37)	0.36 (0.29, 0.40)	0.40 (0.32, 0.47)	0.36 (0.32, 0.47)	0.42 (0.37, 0.53)
	Post	Median (Q1, Q3)	0.43 (0.37, 0.49)	0.43 (0.37, 0.50)	0.38 (0.31, 0.47)	0.40 (0.33, 0.48)	0.42 (0.37, 0.49)	0.39 (0.36, 0.49)	0.45 (0.40, 0.56)
	*p*-value		<0.001*	<0.001*	<0.001*	<0.001*	0.199	0.269	0.232
TR v max, m/s	Pre	Median (Q1, Q3)	2.64 (2.24, 3.17)	2.62 (2.23, 3.14)	2.88 (2.42, 3.26)	2.64 (2.46, 3.14)	2.74 (2.22, 3.07)	2.53 (2.17, 3.01)	3.02 (2.15, 3.44)
	Post	Median (Q1, Q3)	2.20 (1.83, 2.57)	2.10 (1.80, 2.41)	2.40 (2.00, 2.72)	2.50 (1.97, 2.79)	2.59 (2.28, 2.89)	2.30 (1.77, 2.88)	2.49 (1.78, 2.65)
	*p*-value		<0.001*	<0.001*	<0.001*	<0.001*	0.568	0.083	0.066
GLS, %	Pre	Median (Q1, Q3)	18.80 (13.90, 22.30)	20.50 (17.70, 23.30)	8.80 (6.90, 12.55)	12.10 (9.30, 14.20)	16.25 (11.53, 19.58)	20.70 (16.85, 22.65)	18.00 (15.00, 22.10)
	Post	Median (Q1, Q3)	14.00 (10.80, 17.20)	15.20 (12.55, 17.80)	9.40 (7.55, 12.00)	9.60 (8.00, 13.00)	12.40 (8.65, 16.13)	14.40 (12.35, 19.20)	12.40 (10.15, 18.00)
	*p*-value		<0.001*	<0.001*	0.817	<0.001*	0.001*	<0.001*	0.001*
LArS, %	Pre	Median (Q1, Q3)	25.90 (15.90, 35.40)	30.30 (22.55, 38.80)	12.30 (8.70, 16.80)	17.45 (12.93, 24.82)	12.50 (10.25, 18.45)	20.30 (13.15, 33.20)	21.80 (17.50, 32.82)
	Post	Median (Q1, Q3)	20.30 (12.60, 27.60)	23.10 (15.30, 29.60)	15.20 (10.00, 21.50)	14.50 (9.10, 20.67)	10.60 (6.32, 15.63)	18.90 (9.45, 24.55)	22.00 (12.73, 28.60)
	*p*-value		<0.001*	<0.001*	0.087	<0.001*	0.067	0.021*	0.240
LACs, %	Pre	Median (Q1, Q3)	16.80 (10.60, 23.50)	20.40 (15.10, 26.35)	7.70 (4.85, 11.45)	10.40 (6.43, 13.43)	9.75 (8.03, 14.20)	11.10 (5.90, 20.25)	13.70 (9.35, 19.83)
	Post	Median (Q1, Q3)	10.10 (6.50, 14.80)	11.70 (8.35, 16.45)	7.10 (4.45, 9.40)	6.90 (5.13, 10.58)	6.90 (4.73, 9.98)	9.00 (4.55, 13.30)	9.50 (7.00, 15.45)
	*p*-value		<0.001*	<0.001*	0.019*	<0.001*	0.039*	0.026*	0.086
LAbS, %	Pre	Median (Q1, Q3)	7.70 (3.60, 12.60)	8.80 (4.65, 13.70)	3.90 (2.15, 6.80)	7.25 (4.53, 12.55)	2.40 (1.33, 4.35)	8.00 (4.70, 13.10)	8.40 (6.25, 11.30)
	Post	Median (Q1, Q3)	9.10 (4.30, 13.70)	10.00 (5.40, 14.40)	7.40 (3.90, 12.65)	6.35 (3.20, 9.88)	1.60 (1.02, 6.38)	8.20 (3.65, 12.00)	10.40 (5.13, 13.45)
	*p*-value		0.003*	0.013*	<0.001*	0.015*	0.906	0.294	0.695
RVFWS, %	Pre	Median (Q1, Q3)	20.00 (14.50, 25.40)	21.60 (16.08, 26.30)	14.30 (10.70, 19.40)	19.40 (14.70, 24.20)	15.65 (11.83, 20.33)	20.25 (17.08, 25.10)	16.00 (10.07, 24.90)
	Post	Median (Q1, Q3)	14.00 (10.10, 18.30)	15.35 (11.00, 19.30)	11.80 (7.30, 16.10)	12.10 (8.50, 14.80)	10.70 (8.23, 17.15)	15.55 (12.98, 17.20)	9.90 (4.25, 15.77)
	*p*-value		<0.001*	<0.001*	<0.001*	<0.001*	0.011*	0.001*	0.019*

LVFMR, left ventricle functional mitral regurgitation; LAFMR, left atrial functional mitral regurgitation; Q1, quartile 1; Q3, quartile 3; Pre, before mitral valve repair; Post, after mitral valve repair; LVEF, left ventricular ejection fraction; LVESVi, left ventricular end-systolic volume index; LVEDVi, left ventricular end-diastolic volume index; LAVi, left atrial volume index; LVIDd, left ventricular internal dimension in diastole; LVIDs, left ventricular internal dimension in systole; IVSd, interventricular septal thickness in diastole; PWTd, posterior wall thickness at end-diastole; RWT, relative wall thickness; Tr v max, tricuspid regurgitation maximum velocity; GLS, global longitudinal strain; RVFWS, right ventricular free wall strain.

*Statistically significant *p* <0.05.

Analysis of the subgroup with severe MR (532 of 911 patients) demonstrated a similar pattern. Overall, EF decreased from 61.0% to 51.0% (*p* < 0.001), LVEDVi decreased from 79.6 to 53.1 mL/m^2^ (*p* < 0.001), LAVI decreased from 67.7 to 35.6 mL/m^2^ (*p* < 0.001), GLS declined from 20.2% to 14.4% (*p* < 0.001), and LArS decreased from 27.2% to 20.9% (*p* < 0.001). Within this severe MR cohort, changes at 6 months post-surgery by etiology showed that patients with primary MR and rheumatic MR had the greatest reductions in EF (primary MR: 61.8% to 52.0%, *p* < 0.001; rheumatic MR: 59.5% to 50.5%, *p* = 0.002) and GLS (primary MR: 20.7% to 14.9%, *p* < 0.001; rheumatic MR: 21.0% to 13.0%, *p* = 0.002). Primary MR was also associated with a significant reduction in LArS (28.8% to 21.8%, *p* < 0.001), whereas rheumatic MR showed no significant change in LArS (*p* = 0.093). LVFMR and ischemic MR showed no significant changes in EF, GLS, or LArS at 6 months after repair. In contrast, LAFMR demonstrated significant reductions in EF (54.0% to 46.0%, *p* = 0.006), GLS (18.6% to 12.6%, *p* = 0.001), and LArS (11.5% to 8.5%, *p* = 0.004) (Supplementary Table S1).

TR Vmax > 3.4 m/s was considered indicative of a high probability of pulmonary hypertension. In the overall cohort of 911 patients, 19.5% had a TR Vmax > 3.4 m/s. Across etiologies, the highest proportion of patients with TR Vmax > 3.4 m/s was observed in LVFMR (25.0%), followed by Other etiologies (21.4%), primary MR (19.4%), ischemic MR (16.2%), LAFMR (15.6%), and rheumatic MR (13.0%) (Supplementary Figure S1).

Elective surgery accounted for 93% of all MV repairs. The most frequent postprocedural complications were arrhythmias, followed by pericardial effusion and renal failure requiring dialysis, while stroke occurred in only 1.2% of patients. Patients who underwent primary MR demonstrated remarkably low rates of postprocedural complications. Systolic anterior motion developed in only 14 patients, with 13 cases occurring in the primary MR subgroup. Excellent repair outcomes were achieved, with mild or less mitral regurgitation present in 94.3% of the overall cohort and 95.1% of the patients with primary MR. Notably, complete elimination of MR was achieved in 55.3% of patients in the primary MR group. There are no patients with significant mitral stenosis post MV repair (Table 3).

**Table 3:** Perioperative characteristics and clinical outcomes of patients who underwent mitral valve repair based on mechanisms of mitral regurgitation.

Variable		Total *N* = 991	Mechanisms of mitral regurgitation					
			Primary (*N* = 611)	LVFMR (*N* = 124)	Ischemic (*N* = 94)	LAFMR (*N* = 34)	Rheumatic (*N* = 26)	Others (*N* = 22)
Perioperative characteristics								
Procedure urgency, *n* (%)	Elective	847 (93.0)	586 (95.9)	105 (84.7)	81 (86.2)	30 (88.2)	26 (100.0)	19 (86.4)
	Urgent	51 (5.6)	20 (3.3)	15 (12.1)	11 (11.7)	2 (5.9)	0	3 (13.6)
	Emergency	13 (1.4)	5 (0.8)	4 (3.2)	2 (2.1)	2 (5.9)	0	0
Procedure, *n* (%)	MV repair	320 (35.1)	293 (48.0)	4 (3.2)	0	4 (11.8)	11 (42.3)	8 (36.4)
	MV repair + Concomitant valve	107 (11.7)	71 (11.6)	19 (15.3)	0	5 (14.7)	10 (38.5)	2 (9.1)
	MV repair + CABG	249 (27.3)	108 (17.7)	60 (48.4)	75 (79.8)	5 (14.7)	0	1 (4.5)
	MV repair + Others	74 (8.1)	58 (9.5)	3 (2.4)	0	7 (20.6)	2 (7.7)	4 (18.2)
	MV repair + Concomitant valve + CABG	59 (6.5)	21 (3.4)	22 (17.7)	11 (11.7)	1 (2.9)	1 (3.8)	3 (13.6)
	MV repair + Concomitant valve + Others	64 (7.0)	40 (6.5)	11 (8.9)	0	9 (26.5)	2 (7.7)	2 (9.1)
	MV repair + CABG + Others	30 (3.3)	16 (2.6)	3 (2.4)	7 (7.4)	3 (8.8)	0	1 (4.5)
	MV repair + Concomitant valve + CABG + Others	8 (0.9)	4 (0.7)	2 (1.6)	1 (1.1)	0	0	1 (4.5)
EuroSCORE II range	Low risk (<0.81)	314 (34.5)	279 (45.7)	12 (9.7)	6 (6.4)	5 (14.7)	9 (34.6)	3 (13.6)
	Medium risk (0.81–1.22)	186 (20.4)	133 (21.8)	8 (6.5)	21 (22.3)	12 (35.3)	10 (38.5)	2 (9.1)
	Medium to high risk (1.23–2.02)	170 (18.7)	101 (16.5)	27 (21.8)	26 (27.7)	4 (11.8)	5 (19.2)	7 (31.8)
	High risk (2.03–4.11)	146 (16.0)	69 (11.3)	39 (31.5)	23 (24.5)	8 (23.5)	2 (7.7)	5 (22.7)
	Very high risk (≥4.12)	95 (10.4)	29 (4.7)	38 (30.6)	18 (19.1)	5 (14.7)	0	5 (22.7)
EuroSCORE II	Median (Q1, Q3)	1.1 (0.7, 2.1)	0.9 (0.6, 1.5)	2.6 (1.7, 4.6)	1.9 (1.1, 3.5)	1.2 (0.9, 2.7)	0.9 (0.7, 1.4)	1.8 (1.2, 4.0)
Cardiopulmonary bypass time, min	Median (Q1, Q3)	110.0 (82.0, 142.5)	99.0 (75.0, 127.0)	138.0 (116.0, 179.0)	134.0 (114.3, 166.8)	106.5 (75.0, 141.3)	110.0 (93.5, 140.0)	105.0 (78.8, 147.8)
Cross-clamp time, min	Median (Q1, Q3)	83.0 (60.0, 110.0)	74.0 (55.0, 101.0)	108.0 (85.8, 137.0)	105.0 (88.0, 129.0)	75.0 (58.3, 107.3)	93.5 (74.8, 108.5)	85.5 (52.5, 122.3)
Complications post-procedure, *n* (%)	New stroke	11 (1.2)	4 (0.7)	5 (4.0)	1 (1.1)	1 (2.9)	0	0
	Renal failure requiring dialysis	51 (5.6)	13 (2.1)	18 (14.5)	13 (13.8)	2 (5.9)	1 (3.8)	4 (18.2)
	Pulmonary complication	46 (5.0)	18 (2.9)	13 (10.5)	7 (7.4)	6 (17.6)	1 (3.8)	1 (4.5)
	GI complication	17 (1.9)	7 (1.1)	5 (4.0)	5 (5.3)	0	0	0
	Surgical site infection	17 (1.9)	6 (1.0)	5 (4.0)	5 (5.3)	1 (2.9)	0	0
	New heart failure	12 (1.3)	6 (1.0)	2 (1.6)	3 (3.2)	1 (2.9)	0	0
	Period MI	6 (0.7)	1 (0.2)	2 (1.6)	3 (3.2)	0	0	0
	Arrythmias	256 (28.1)	147 (24.1)	46 (37.1)	37 (39.4)	15 (44.1)	6 (23.1)	5 (22.7)
	Pericardial effusion	74 (8.1)	47 (7.7)	8 (6.5)	9 (9.6)	6 (17.6)	1 (3.8)	3 (13.6)
	Pleural effusion	13 (1.4)	6 (1.0)	4 (3.2)	2 (2.1)	0	0	1 (4.5)
	Fever	32 (3.5)	11 (1.8)	9 (7.3)	7 (7.4)	3 (8.8)	0	2 (9.1)
Clinical outcomes								
Systolic anterior motion postop, *n* (%)		14 (1.5)	13 (2.1)	0	0	0	1 (4.0)	0
Residual MR postop, *n* (%)	None	484 (53.2)	338 (55.3)	66 (53.2)	42 (44.7)	20 (58.8)	12 (48.0)	6 (27.3)
	Trivial	279 (30.7)	194 (31.8)	35 (28.2)	25 (26.6)	8 (23.5)	6 (24.0)	11 (50.0)
	Mild	95 (10.4)	49 (8.0)	16 (12.9)	15 (16.0)	3 (8.8)	7 (28.0)	5 (22.7)
	Moderate	45 (4.9)	24 (3.9)	6 (4.8)	12 (12.8)	3 (8.8)	0	0
	Severe	7 (0.8)	6 (1.0)	1 (0.8)	0	0	0	0
Length of stay postop, day	Median (Q1, Q3)	8.0 (6.0, 11.0)	7.0 (6.0, 9.0)	9.0 (7.0, 15.0)	10.0 (7.0, 14.0)	10.5 (7.0, 16.8)	7.0 (6.0, 9.3)	11.5 (7.0, 15.0)
Total hospital stay, day	Median (Q1, Q3)	11.0 (9.0, 16.0)	10.0 (9.0, 13.0)	16.0 (10.3, 24.0)	15.0 (10.0, 22.0)	14.5 (10.0, 22.3)	10.0 (9.0, 12.3)	17.5 (10.8, 26.3)
In-hospital outcome, *n* (%)	Alive	862 (94.6)	599 (98.0)	106 (85.5)	81 (86.2)	30 (88.2)	26 (100.0)	20 (90.9)
	Died	49 (5.4)	12 (2.0)	18 (14.5)	13 (13.8)	4 (11.8)	0	2 (9.1)
Comparison of all-cause mortality								
All-cause mortality postop, *n* (%)	Within 30 days^a^	50 (5.5)	13 (2.1)	19 (15.3)	13 (13.8)	3 (8.8)	0	2 (9.1)
	Within 6 months^a^	73 (8.0)	22 (3.6)	27 (21.8)	17 (18.1)	5 (14.7)	0	2 (9.1)
	Within 12 months^a^	80 (8.8)	27 (4.4)	29 (23.4)	17 (18.1)	5 (14.7)	0	2 (9.1)
	Overall ^b^	176 (19.3)	72 (11.8)	53 (42.7)	33 (35.1)	14 (41.2)	0	4 (18.2)

LVFMR, left ventricle functional mitral regurgitation; LAFMR, left atrial functional mitral regurgitation; MV, mitral valve; CABG,; GI, gastrointestinal; MI, myocardial infarction; postop, postoperatively; Q1, quartile 1; Q3, quartile 3.

a Patients were considered alive unless mortality was reported to either our hospital or the Malaysia National Registration Department, regardless of continued follow-up at other health facilities or loss to follow-up. Bias may occur, particularly for foreign patients and those lost to follow-up.

b Overall postoperative mortality accounts for deaths from the postoperative period to the last follow-up, with a median duration post-procedure of 51.0 (17.3, 79.9). Bias may have occurred due to differences in follow-up duration and reliance on retrospective patient follow-up data.

The overall in-hospital mortality rate was 5.4% (49 patients), with marked variation across the subgroups. The LVFMR group exhibited the highest in-hospital mortality (14.5%), followed by the ischemic (13.8%) and LAFMR (11.8%) groups. In contrast, the primary group demonstrated a notably lower in-hospital mortality rate of 2.0%, whereas the rheumatic group experienced no deaths. The others group had an intermediate mortality rate of 9.1%. At the 6-month follow-up, the cumulative mortality increased to 8.0% (73 patients) in the overall cohort, with the most pronounced increase observed in the LVFMR group, where mortality increased to 21.8%, followed by the ischemic group at 18.1% and the LAFMR group at 14.7%. The primary group maintained a low mortality rate of 3.6%, whereas the rheumatic group continued to have no deaths. Twelve-month mortality data showed minimal progression from the 6-month outcomes, with the overall rate increasing marginally to 8.8% (80 patients). The LVFMR group continued to demonstrate the highest mortality rate (23.4%), whereas the ischemic and LAFMR groups maintained their previous mortality rates at 18.1% and 14.7%, respectively. The primary group showed a slight increase in mortality to 4.4%, whereas the rheumatic group sustained a zero-mortality rate. Long-term follow-up at 51 months (median follow-up range, 17.3–79.9 months) revealed substantial increases in mortality across all groups except the rheumatic group, with overall mortality increasing to 19.3% (176 patients). The LVFMR group demonstrated the highest long-term mortality rate (42.7%), followed closely by the LAFMR (41.2%) and ischemic groups (35.1%). The primary group, while maintaining the lowest mortality rate among the groups with reported deaths, showed an increased mortality at 11.8%. The others group demonstrated an intermediate long-term mortality rate of 18.2%, whereas the rheumatic group maintained zero mortality throughout the follow-up period.

For patients with severe MR, overall in-hospital mortality was slightly lower than in the entire cohort (3.2% vs. 5.4%). In-hospital mortality for primary MR was similar between the severe and overall groups (1.8% vs. 2.0%). The highest in-hospital mortality was observed in LAFMR (20%), followed by LVFMR (12.0%) and ischemic MR (11.1%). At a median follow-up of 51 months (4.3 years), overall mortality was also lower in the severe MR group compared with the overall cohort (16.0% vs. 19.3%). Mortality in primary MR remained similar between the severe and overall groups at 51 months (12.4% vs. 11.8%). The three etiologies with the highest overall mortality at 51 months were the same in both the severe and overall groups: LVFMR (severe 56.0%, overall 42.7%), LAFMR (severe 46.7%, overall 41.2%), and ischemic MR (severe 33.3%, overall 35.1%) (Supplementary Table S2).

**Figure 5:**
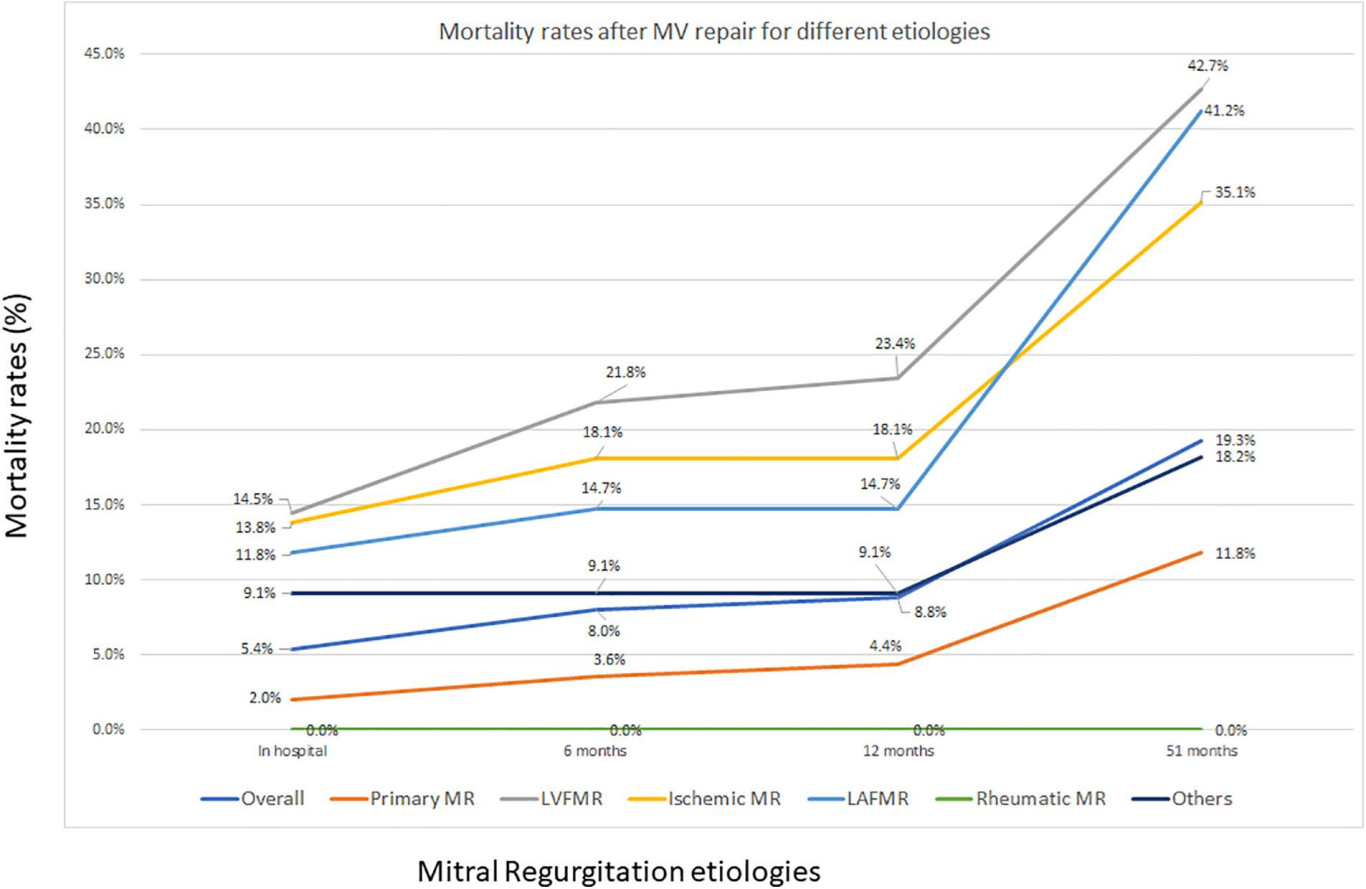
Mortality rates after mitral valve repair. Mortality is worst when the MR mechanisms is the left ventricle and/or the left atrium and is the best when the mechanism is the leaflet itself. MR, mitral regurgitation; LVFMR, left ventricle functional mitral regurgitation; LAFMR, left atrial functional mitral regurgitation.

**Figure 6:**
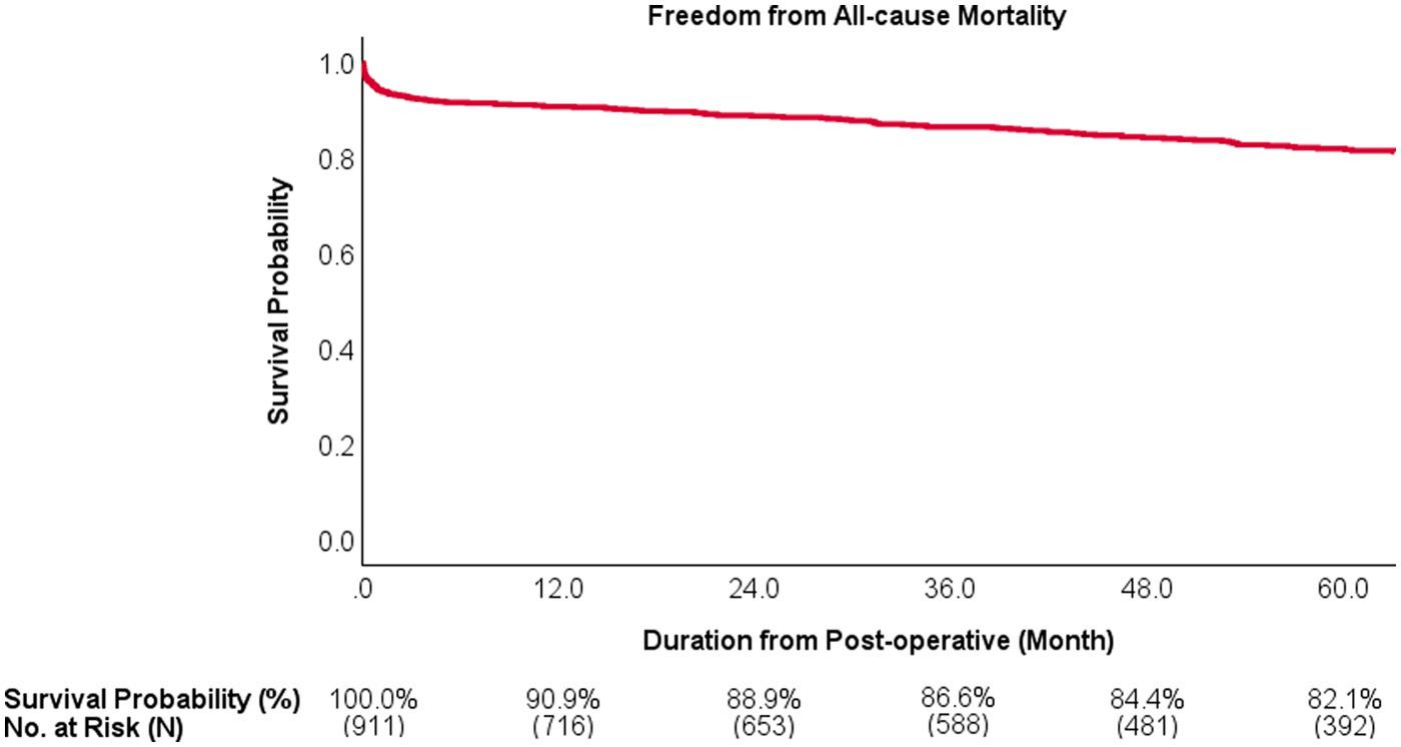
Freedom from all-cause mortality overall. At 5 years, the survival probability is 82.1% overall.

**Figure 7:**
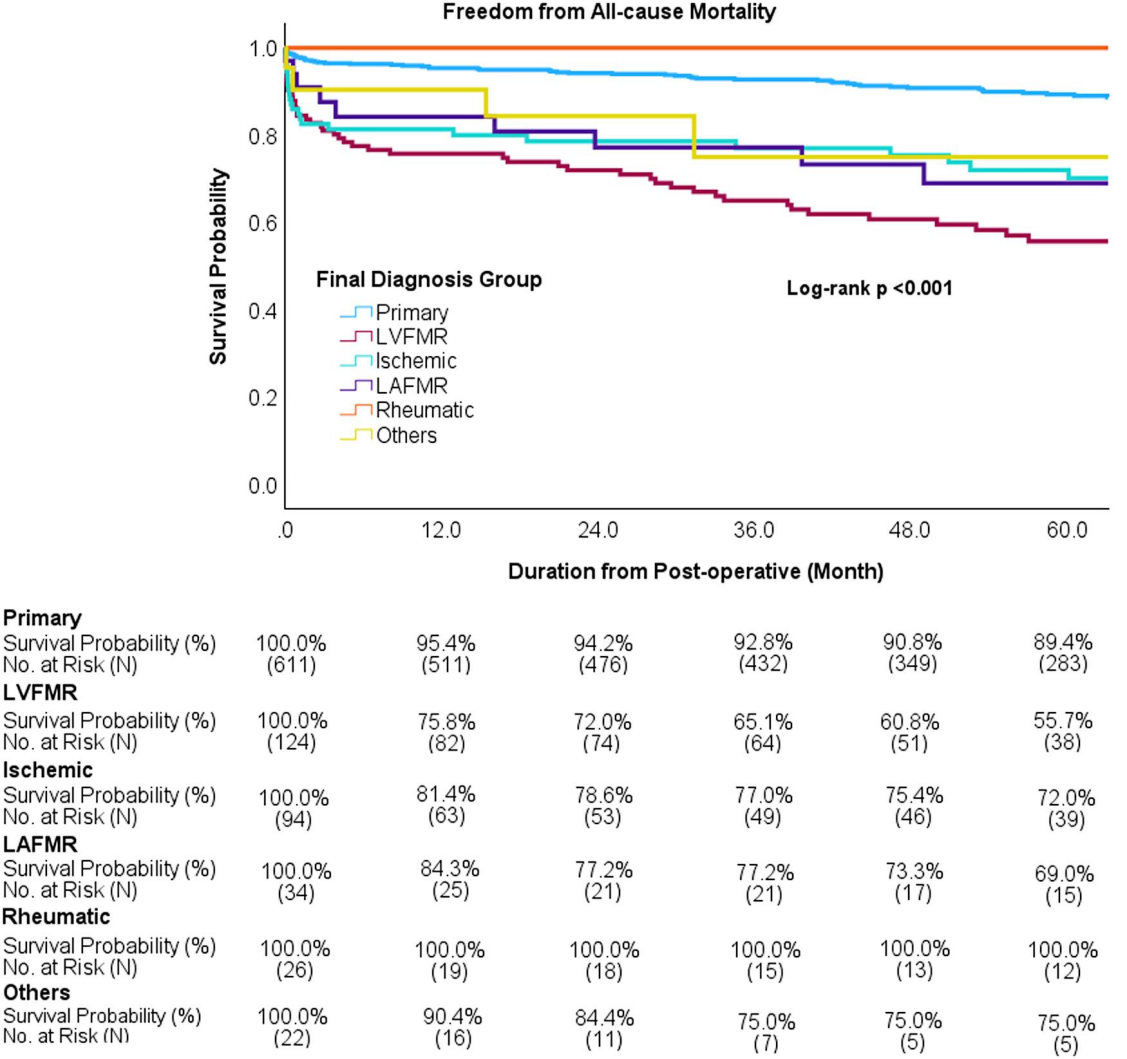
Freedom from all-cause mortality by group. At 5 years, the top three worst survival rates are observed in the LVFMR, LAFMR, and ischemic MR groups. LVFMR, left ventricle functional mitral regurgitation; LAFMR, left atrial functional mitral regurgitation; MR, mitral regurgitation.

The temporal mortality pattern revealed that while early mortality risk stabilized between 6-months and 12-months post-intervention, there was a substantial increase in mortality risk during the extended follow-up, which was particularly pronounced in the high-risk subgroups (Table 3; Figure 5). Kaplan–Meier survival analysis at 5 years demonstrated 82.1% overall freedom from all-cause mortality. Subgroup analysis revealed a clear hierarchy of survival outcomes, with the LVFMR group experiencing the poorest survival at 5 years, followed by the LAFMR, ischemic, others, and primary groups, whereas the rheumatic group maintained 100% survival (Figures 6, 7).

## 4 Discussion

The findings of this study demonstrated significant differences in outcomes following MV repair across various etiologies of MR. There was a clear stratification of mortality risk across different patient subgroups, with patients with LVFMR, LAFMR, and ischemic MR exhibiting particularly increased mortality rates during the early post-intervention period and throughout the long-term follow-up. The primary MR group consistently maintained the lowest mortality rates among the etiologies with reported deaths, whereas the rheumatic group demonstrated exceptional survival, with zero mortality throughout the entire study period. A similar pattern was observed in the subgroup analysis restricted to patients with severe MR. One important confounding factor underlying the observed zero mortality in Rheumatic MR is their substantially younger age compared with the other etiologic groups. Age is a major determinant of operative and long-term risk, and this age difference likely contributed significantly to the absence of mortality in this cohort. Stratifying patients into primary, functional (LVFMR and LAFMR), ischemic, and rheumatic groups enabled comprehensive evaluation of structural and functional cardiac changes post-repair as well as their possible impact on short-term and medium-term mortalities (7–9). Since the advent of the Carpentier repair technique, numerous improvements in the surgical approach have been introduced (10–14), establishing MV repair as the current gold standard for primary MR. While many studies have focused on primary MR, this is the first comprehensive analysis of outcomes, echocardiographic changes, and various strain parameters (excluding right atrial strain) across diverse etiologies (15–17).

Significant improvements in cardiac dimensions and volumes, as evidenced by the reductions in the LVESVi, LVEDVi, and LAVI, demonstrate that surgical intervention effectively alleviates the hemodynamic burden imposed by MR. However, the concurrent decline in myocardial function, particularly the reductions in the EF, GLS, and LArS observed in most groups (except the LVFMR group), revealed a complex interplay between structural correction and myocardial recovery. It is well recognized that pericardial incision during cardiac surgery causes a transient reduction in both left and right ventricular function, which typically recovers within 6 months postoperatively. In addition, the Frank–Starling mechanism affects contractility when preload decreases after surgery. Together, these factors can confound the assessment of intrinsic myocardial recovery following MV repair (18). The LVFMR group exhibited the most intriguing changes, being the only subgroup demonstrating improvements in the EF, GLS, and LArS, along with the smallest reduction in RVFWS compared with all other groups. Paradoxically, despite these functional improvements, the LVFMR group had the highest mortality rate at all the time points. This discrepancy suggests that while hemodynamic parameters may improve following repair, myocardial recovery may be delayed or insufficient in certain subgroups, particularly in those with preexisting myocardial and left atrial dysfunction. Whether these changes in echocardiographic and strain parameters correlate with the clinical outcomes remains uncertain and warrants further investigation. Although prior studies have examined individual strain values, none of them have comprehensively evaluated all strain parameters across the various MR etiologies (19–23).

The substantial variation in short-term and long-term mortality rates among the subgroups underscores the critical need for etiology-specific risk stratification. The primary and rheumatic MR groups demonstrated low in-hospital and medium-term mortalities, with the rheumatic group achieving zero mortality, contrasting sharply with the high mortality rates observed in the LVFMR, LAFMR, and ischemic MR groups. These findings suggest that in conditions characterized by prominent myocardial or atrial dysfunction, addressing MR alone is insufficient and that additional therapeutic measures targeting the underlying pathophysiology are necessary to improve survival outcomes (21). Notably, mortality outcomes for primary MR in this cohort are comparable to, and in some studies superior to, those reported in Western and more developed Asia-Pacific countries (15, 16, 24).

The results of this study further emphasize the critical importance of patient selection and the optimal timing of intervention. For the primary and rheumatic MR groups, the median preoperative EF approached 60%, and the median left ventricular internal diameter in systole remained below 4 cm, indicating that repair was performed before the development of irreversible left ventricular dysfunction. The excellent outcomes observed in these groups highlight that early and appropriately timed interventions in patients with less complex pathophysiology are likely to yield favorable results (25). Conversely, the inferior outcomes in the LVFMR, LAFMR, and ischemic MR groups necessitate enhanced preoperative risk assessment and development of tailored therapeutic strategies that address the multifactorial nature of these conditions. The zero-mortality achieved in the rheumatic group is particularly reassuring, and the echocardiographic and strain patterns, which are remarkably similar to those observed in the primary group, further support the adoption of similar surgical timing approaches for rheumatic and primary MR (26–28).

This study has several important limitations. As a retrospective, single-center analysis, any observed associations cannot be interpreted as causal. The demographic and baseline characteristics of the etiologic groups were not propensity score–matched. Given the substantial differences in age, concomitant procedures, and comorbidities between groups, it was not feasible to fully adjust for age or other potential confounders. For example, all patients with ischemic MR underwent concomitant CABG, whereas only 3.8% of the rheumatic group and 24.4% of the primary MR group had CABG. In addition, the relatively small sample sizes in the rheumatic (*n* = 26) and LAFMR (*n* = 34) subgroups further limit the robustness of adjusted analyses. Future studies should examine each etiology in greater detail and incorporate larger cohorts from multiple centers to address these limitations.

### 4.1 Conclusions

This study confirms the efficacy of MV repair in reducing MR and improving cardiac hemodynamics across all patient groups, with excellent procedural success rates. The primary determinant of outcomes was the underlying MR mechanism, with primary and rheumatic MR demonstrating the most favorable results, whereas the LVFMR, LAFMR, and ischemic MR groups exhibited significantly worse outcomes. These findings highlight the critical importance of etiology-specific approaches and warrant further research into comprehensive management strategies that integrate mechanical correction and optimized medical therapy, particularly for patients in whom leaflet pathology is not the primary underlying mechanism driving MR.

## Data Availability

The raw data supporting the conclusions of this article will be made available by the authors, without undue reservation.
