# Supplementary material for: Changes in echocardiographic parameters and strain and outcomes following mitral valve repair: insight from new mitral regurgitation classifications
Source: Front Cardiovasc Med. 2026 Jan 12;12:1666071. doi: 10.3389/fcvm.2025.1666071 (PMC12832881; doi:10.3389/fcvm.2025.1666071)
Supplement: Supplementary file 1 [file Supplementaryfile1.docx]

**Supplementary Table 1** Echocardiography and strain analysis pre- and post-mitral valve repair according to different mechanisms of severe MR.

| **Variables** | | | **Total N = 532** | **Mechanisms of Severe Mitral Regurgitation** | | | | | |
| --- | --- | --- | --- | --- | --- | --- | --- | --- | --- |
|  |  |  |  | **Primary  (N = 445)** | **LVFMR (N = 25)** | **Ischemic (N =18)** | **LAFMR (N = 15)** | **Rheumatic (N = 18)** | **Others (N = 11)** |
| LVEF; % | Pre | Median (Q1, Q3) | 61.00 (55.00, 65.00) | 61.75 (57.00, 65.20) | 32.00 (28.05, 37.05) | 38.00 (35.13, 43.38) | 54.00 (51.50, 57.00) | 59.50 (54.25, 66.78) | 61.00 (54.00, 66.00) |
|  | Post | Median (Q1, Q3) | 51.00 (45.00, 56.00) | 52.00 (46.00, 57.00) | 30.00 (23.50, 42.50) | 40.00 (35.00, 45.50) | 46.00 (43.00, 52.00) | 50.50 (43.00, 55.00) | 46.00 (35.00, 55.00) |
|  | *p-value* | | <0.001* | <0.001* | 0.590 | 0.442 | 0.006* | 0.002* | 0.010* |
| LVESVi; mL/m² | Pre | Median (Q1, Q3) | 31.57 (24.70, 42.14) | 30.31 (24.18, 39.15) | 66.49 (54.72, 89.51) | 48.10 (33.36, 63.25) | 33.33 (28.55, 39.54) | 31.44 (20.10, 44.05) | 35.24 (25.21, 55.38) |
|  | Post | Median (Q1, Q3) | 25.08 (19.63, 33.92) | 24.27 (19.38, 31.87) | 46.70 (35.74, 65.40) | 40.89 (29.69, 52.40) | 30.27 (21.08, 38.54) | 24.99 (17.74, 47.88) | 23.55 (14.10, 37.31) |
|  | *p-value* | | <0.001* | <0.001* | 0.010* | 0.408 | 0.196 | 0.267 | 0.285 |
| LVEDVi; mL/m² | Pre | Median (Q1, Q3) | 79.60 (63.69, 97.56) | 79.44 (63.88, 96.30) | 98.61 (82.68, 130.80) | 82.06 (60.44, 104.27) | 72.23 (61.65, 84.98) | 74.63 (54.48, 97.38) | 81.37 (56.91, 112.75) |
|  | Post | Median (Q1, Q3) | 53.05 (43.72, 64.89) | 52.48 (43.26, 62.87) | 69.27 (54.93, 86.86) | 69.06 (59.92, 87.19) | 51.34 (42.21, 71.33) | 50.69 (36.97, 78.30) | 50.08 (33.00, 59.76) |
|  | *p-value* | | <0.001* | <0.001* | 0.010* | 0.121 | 0.009* | 0.004* | 0.009* |
| LAVi; mL/m² | Pre | Median (Q1, Q3) | 67.69 (51.61, 93.16) | 66.31 (51.20, 90.59) | 63.08 (46.88, 81.68) | 61.06 (40.95, 74.84) | 127.53 (72.55, 194.35) | 112.86 (74.40, 151.42) | 82.95 (70.52, 108.02) |
|  | Post | Median (Q1, Q3) | 35.55 (27.99, 48.01) | 34.23 (27.20, 46.35) | 39.47 (29.81, 58.76) | 41.19 (37.46, 50.54) | 83.12 (44.31, 127.41) | 54.89 (45.81, 68.32) | 30.95 (26.55, 88.93) |
|  | *p-value* | | <0.001* | <0.001* | <0.001* | 0.044* | 0.009* | <0.001* | 0.021* |
| LVIDd; cm | Pre | Median (Q1, Q3) | 5.80 (5.32, 6.21) | 5.76 (5.30, 6.16) | 6.26 (5.76, 6.85) | 6.06 (5.67, 6.40) | 5.46 (5.06, 6.35) | 5.67 (5.34, 6.58) | 5.90 (4.73, 6.40) |
|  | Post | Median (Q1, Q3) | 4.70 (4.30, 5.20) | 4.70 (4.30, 5.10) | 5.50 (5.00, 5.90) | 5.55 (4.85, 5.95) | 5.10 (4.35, 5.70) | 4.64 (3.93, 5.25) | 4.40 (4.00, 4.90) |
|  | *p-value* | | <0.001* | <0.001* | <0.001* | 0.004* | 0.019* | <0.001* | 0.014* |
| LVIDs; cm | Pre | Median (Q1, Q3) | 3.72 (3.29, 4.20) | 3.66 (3.22, 4.10) | 5.35 (4.70, 5.81) | 4.92 (4.23, 5.32) | 4.00 (3.57, 4.42) | 3.70 (3.44, 4.68) | 4.00 (3.00, 4.10) |
|  | Post | Median (Q1, Q3) | 3.25 (2.90, 3.80) | 3.20 (2.90, 3.60) | 4.30 (3.95, 5.15) | 4.40 (3.65, 5.10) | 3.60 (3.05, 4.15) | 3.40 (2.76, 4.15) | 3.50 (3.01, 4.30) |
|  | *p-value* | | <0.001* | <0.001* | <0.001* | 0.026* | 0.133 | 0.043* | 0.965 |
| IVSd; cm | Pre | Median (Q1, Q3) | 1.07 (0.94, 1.20) | 1.09 (0.96, 1.20) | 1.00 (0.94, 1.18) | 1.09 (0.98, 1.22) | 1.10 (0.88, 1.19) | 0.92 (0.79, 1.01) | 1.20 (0.83, 1.30) |
|  | Post | Median (Q1, Q3) | 1.00 (0.90, 1.20) | 1.00 (0.90, 1.19) | 1.00 (0.80, 1.25) | 1.08 (0.83, 1.28) | 1.10 (0.90, 1.20) | 0.90 (0.80, 1.10) | 0.91 (0.80, 1.50) |
|  | *p-value* | | 0.001* | <0.001* | 0.962 | 0.712 | 0.894 | 0.552 | 0.306 |
| PWTd; cm | Pre | Median (Q1, Q3) | 1.03 (0.90, 1.19) | 1.04 (0.90, 1.20) | 0.95 (0.82, 1.08) | 0.92 (0.79, 1.12) | 1.10 (0.79, 1.19) | 0.95 (0.89, 1.23) | 1.20 (1.00, 1.35) |
|  | Post | Median (Q1, Q3) | 1.00 (0.90, 1.19) | 1.00 (0.90, 1.20) | 1.10 (0.90, 1.30) | 0.90 (0.83, 1.02) | 1.00 (0.90, 1.14) | 0.98 (0.88, 1.12) | 1.10 (0.90, 1.20) |
|  | *p-value* | | 0.837 | 0.977 | 0.007* | 0.802 | 0.875 | 0.449 | 0.130 |
| RWT | Pre | Median (Q1, Q3) | 0.36 (0.30, 0.43) | 0.36 (0.30, 0.43) | 0.29 (0.26, 0.34) | 0.33 (0.27, 0.36) | 0.35 (0.32, 0.45) | 0.36 (0.31, 0.47) | 0.37 (0.34, 0.57) |
|  | Post | Median (Q1, Q3) | 0.44 (0.37, 0.50) | 0.44 (0.38, 0.50) | 0.38 (0.31, 0.47) | 0.33 (0.30, 0.45) | 0.42 (0.37, 0.48) | 0.40 (0.37, 0.49) | 0.45 (0.39, 0.60) |
|  | *p-value* | | <0.001* | <0.001* | <0.001* | 0.049* | 0.382 | 0.248 | 0.374 |
| TR v max; m/s | Pre | Median (Q1, Q3) | 2.81 (2.29, 3.30) | 2.81 (2.31, 3.30) | 2.74 (2.15, 3.03) | 3.36 (2.57, 3.84) | 2.65 (2.28, 3.11) | 2.41 (2.17, 3.25) | 3.03 (2.40, 3.34) |
|  | Post | Median (Q1, Q3) | 2.15 (1.80, 2.50) | 2.10 (1.80, 2.40) | 2.36 (1.85, 2.82) | 2.55 (2.09, 2.77) | 2.59 (2.25, 2.85) | 2.45 (1.64, 2.98) | 2.15 (1.32, 2.80) |
|  | *p-value* | | <0.001* | <0.001* | 0.209 | 0.019* | 0.248 | 0.125 | 0.046* |
| GLS; % | Pre | Median (Q1, Q3) | 20.20 (16.90, 23.30) | 20.70 (17.90, 23.63) | 10.20 (7.77, 14.33) | 11.60 (8.65, 14.90) | 18.60 (15.10, 22.20) | 21.00 (17.40, 22.65) | 18.00 (13.60, 24.90) |
|  | Post | Median (Q1, Q3) | 14.35 (11.63, 17.60) | 14.85 (12.30, 17.90) | 9.80 (7.73, 12.10) | 11.10 (8.50, 13.20) | 12.60 (8.80, 16.90) | 13.00 (11.80, 18.30) | 10.20 (7.70, 16.30) |
|  | *p-value* | | <0.001* | <0.001* | 0.381 | 0.449 | 0.001* | 0.002* | 0.004* |
| LArS; % | Pre | Median (Q1, Q3) | 27.20 (18.40, 35.50) | 28.75 (21.55, 36.93) | 13.60 (8.90, 21.08) | 16.10 (7.80, 19.10) | 11.50 (10.10, 17.00) | 19.80 (12.80, 30.90) | 28.90 (16.60, 33.65) |
|  | Post | Median (Q1, Q3) | 20.90 (12.70, 28.40) | 21.80 (13.98, 29.35) | 14.10 (9.95, 22.95) | 11.90 (8.15, 20.40) | 8.50 (5.10, 14.90) | 16.90 (8.55, 22.70) | 14.45 (7.85, 25.28) |
|  | *p-value* | | <0.001* | <0.001* | 0.833 | 0.758 | 0.004* | 0.093 | 0.022* |
| LACs; % | Pre | Median (Q1, Q3) | 18.50 (13.20, 24.80) | 20.30 (15.20, 25.63) | 8.75 (4.75, 13.43) | 9.40 (6.45, 13.00) | 9.60 (8.10, 10.80) | 9.70 (6.60, 19.55) | 16.65 (10.60, 27.08) |
|  | Post | Median (Q1, Q3) | 10.40 (6.90, 14.70) | 10.90 (7.60, 15.50) | 7.05 (4.23, 11.40) | 6.90 (5.15, 12.25) | 6.50 (2.90, 9.90) | 8.60 (4.90, 12.10) | 8.25 (4.63, 13.58) |
|  | *p-value* | | <0.001* | <0.001* | 0.104 | 0.523 | 0.047* | 0.102 | 0.028* |
| LAbS; % | Pre | Median (Q1, Q3) | 7.50 (3.50, 11.70) | 7.95 (4.00, 12.20) | 5.30 (2.18, 8.13) | 4.40 (1.90, 8.10) | 2.00 (1.20, 4.50) | 6.30 (3.75, 12.45) | 6.50 (3.82, 11.75) |
|  | Post | Median (Q1, Q3) | 9.50 (4.00, 14.20) | 9.90 (5.27, 14.60) | 6.60 (3.38, 11.50) | 5.00 (1.55, 9.20) | 1.20 (1.00, 2.20) | 8.20 (2.65, 11.75) | 5.60 (1.95, 10.45) |
|  | *p-value* | | <0.001* | <0.001* | 0.026* | 0.906 | 0.300 | 0.653 | 0.575 |
| RVFWS; % | Pre | Median (Q1, Q3) | 20.20 (15.00, 25.50) | 20.80 (15.55, 25.70) | 16.50 (10.18, 23.05) | 18.70 (13.75, 25.55) | 17.80 (12.60, 22.90) | 20.00 (14.95, 22.40) | 13.10 (5.90, 22.80) |
|  | Post | Median (Q1, Q3) | 14.10 (10.20, 18.10) | 14.40 (10.80, 18.70) | 10.50 (5.45, 14.95) | 12.10 (9.10, 15.60) | 10.00 (8.20, 17.30) | 15.00 (11.55, 17.10) | 8.90 (3.50, 17.40) |
|  | *p-value* | | <0.001* | <0.001* | 0.002* | 0.003* | 0.025* | 0.011* | 0.168 |

**Supplementary Table 2** Mortality rate among different mechanisms in severe MR.

|  |  | **Total N = 532** | **Primary  (N = 445)** | **LVFMR (N = 25)** | **Ischemic (N =18)** | **LAFMR (N = 15)** | **Rheumatic (N = 18)** | **Others (N = 11)** |
| --- | --- | --- | --- | --- | --- | --- | --- | --- |
| **Clinical Outcomes** | | | | | | | | |
| In-hospital Outcome; n (%) | Alive | 515 (96.8) | 437 (98.2) | 22 (88.0) | 16 (88.9) | 12 (80.0) | 18 (100.0) | 10 (90.9) |
|  | Died | 17 (3.2) | 8 (1.8) | 3 (12.0) | 2 (11.1) | 3 (20.0) | 0 | 1 (9.1) |
| **All-cause Mortality Comparison (severe MR)** | | | | | | | | |
| All-cause Mortality Post-operative, n (%) | Within 30 days | 17 (3.2) | 9 (2.0) | 3 (12.0) | 2 (11.1) | 2 (13.3) | 0 | 1 (9.1) |
|  | Within 6 months | 29 (5.5) | 15 (3.4) | 7 (28.0) | 3 (16.7) | 3 (20.0) | 0 | 1 (9.1) |
|  | Within 12 months | 33 (6.2) | 19 (4.3) | 7 (28.0) | 3 (16.7) | 3 (20.0) | 0 | 1 (9.1) |
|  | Overall | 85 (16.0) | 55 (12.4) | 14 (56.0) | 6 (33.3) | 7 (46.7) | 0 | 3 (27.3) |

**Supplementary Figure 1.**  TR v max ≥ 3.4m/s vs ≤ 3.4m/s in different mechanisms of MR.
